# Maternal smoking impacts key biological pathways in newborns through epigenetic modification *in Utero*

**DOI:** 10.1186/s12864-016-3310-1

**Published:** 2016-11-25

**Authors:** Daniel M. Rotroff, Bonnie R. Joubert, Skylar W. Marvel, Siri E. Håberg, Michael C. Wu, Roy M. Nilsen, Per M. Ueland, Wenche Nystad, Stephanie J. London, Alison Motsinger-Reif

**Affiliations:** 1 Bioinformatics Research Center, North Carolina State University, Raleigh, NC USA; 2 Department of Statistics, North Carolina State University, Raleigh, NC USA; 3 Division of Intramural Research, National Institute of Environmental Health Sciences, National Institutes of Health, Department of Health and Human Services, PO Box 12233, MD A3-05, Research Triangle Park, NC 27709 USA; 4 Norwegian Institute of Public Health, Oslo, Norway; 5 Public Health Sciences Division, Fred Hutchinson Cancer Research Center, Seattle, WA USA; 6 Centre for Clinical Research, Haukeland University Hospital, Bergen, Norway; 7 Department of Clinical Science, University of Bergen, Bergen, Norway; 8 Laboratory of Clinical Biochemistry, Haukeland University Hospital, Bergen, Norway; 9 Center for Comparative Medicine and Translational Research, North Carolina State University, Raleigh, NC USA

**Keywords:** Smoking, Epigenetics, Pathway analysis, Cancer, *In utero*

## Abstract

**Background:**

Children exposed to maternal smoking during pregnancy exhibit increased risk for many adverse health effects. Maternal smoking influences methylation in newborns at specific CpG sites (CpGs). Here, we extend evaluation of individual CpGs to gene-level and pathway-level analyses among 1062 participants in the Norwegian Mother and Child Cohort Study (MoBa) using the Illumina 450 K platform to measure methylation in newborn DNA and maternal smoking in pregnancy, assessed using the biomarker, plasma cotinine. We used novel implementations of bioinformatics tools to collapse epigenome-wide methylation data into gene- and pathway-level effects to test whether exposure to maternal smoking *in utero* differentially methylated CpGs in genes enriched in biologic pathways. Unlike most pathway analysis applications, our approach allows replication in an independent cohort.

**Results:**

Data on 485,577 CpGs, mapping to a total of 20,199 genes, were used to create gene scores that were tested for association with maternal plasma cotinine levels using Sequence Kernel Association Test (SKAT), and 15 genes were found to be associated (*q* < 0.25). Six of these 15 genes (*GFI1*, *MYO1G*, *CYP1A1*, *RUNX1*, *LCTL*, and *AHRR)* contained individual CpGs that were differentially methylated with regards to cotinine levels (*p* < 1.06 × 10^−7^). Nine of the 15 genes (*FCRLA*, *MIR641*, *SLC25A24, TRAK1*, *C1orf180*, *ITLN2*, *GLIS1*, *LRFN1*, and *MIR451)* were associated with cotinine at the gene-level (*q* < 0.25) but had no genome-wide significant individual CpGs (*p* > 1.06 × 10^−7^). Pathway analyses using gene scores resulted in 51 significantly associated pathways, which we tested for replication in an independent cohort (*q* < 0.05). Of those 32 replicated in an independent cohort, which clustered into six groups. The largest cluster consisted of pathways related to cancer, cell cycle, ERα receptor signaling, and angiogenesis. The second cluster, organized into five smaller pathway groups, related to immune system function, such as T-cell regulation and other white blood cell related pathways.

**Conclusions:**

Here we use novel implementations of bioinformatics tools to determine biological pathways impacted through epigenetic changes *in utero* by maternal smoking in 1062 participants in the MoBa, and successfully replicate these findings in an independent cohort. The results provide new insight into biological mechanisms that may contribute to adverse health effects from exposure to tobacco smoke *in utero*.

**Electronic supplementary material:**

The online version of this article (doi:10.1186/s12864-016-3310-1) contains supplementary material, which is available to authorized users.

## Background

Although many adverse effects of maternal smoking on offspring have been well identified, little is known about the underlying biological mechanisms. [1, 2] One proposed mechanism for how *in utero* exposure to tobacco smoke may impact health is through epigenetic effects including DNA methylation. Previously, Joubert et al. collected genome-wide methylation data from 1062 MoBa mother-offspring pairs and demonstrated that maternal smoking, assessed objectively by cotinine levels, is significantly associated with 1) differential DNA methylation in genes involved in metabolism of tobacco smoke compounds, and 2) novel genes involved in diverse developmental processes not previously linked to tobacco response [3]. These findings have since been widely replicated [3–6].

It has been recognized that genome wide association studies, using single nucleotide polymorphisms, that rely on single locus variation explain little of the overall heritability of complex traits [7, 8]. While there are many potential sources of this “missing heritability”, single locus analysis typically ignores a large number of loci with moderate effects, due to stringent significance thresholds. Gene-based association analysis takes a gene as basic unit for association analysis. As this method can combine genetic information given by all the markers in a gene, it can obtain more informative results and increase the capability of finding novel genes and gene sets. This method has been used as a novel complement method for SNP-based GWAS in identifying disease susceptibility genes [9, 10], and we extend such an approach to methylation data here.

**Fig. 1:**
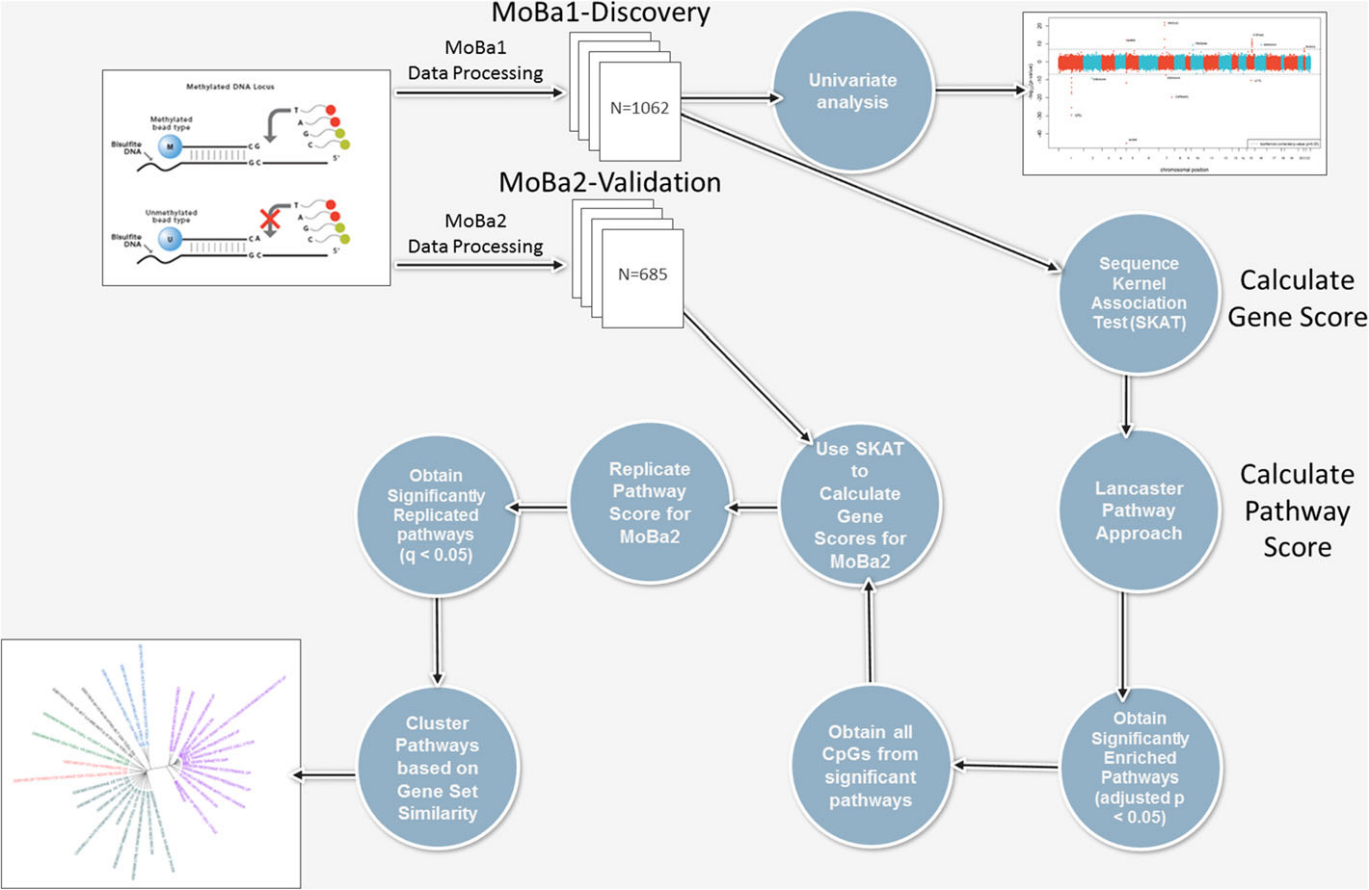
Analysis workflow collapsing individual CpG data into gene- and pathway-level scores, and replication of findings

Additionally, To investigate the biological processes (i.e. pathways) impacted by maternal smoking during pregnancy and associated altered fetal methylation, we performed gene set/pathway analysis to further dissect the biological impact of maternal smoking. We applied a novel approach that combines analysis tools for collapsing epigenome-wide methylation data into gene- and pathway-based effects (Fig. 1). Pathway analysis combines significant genes into sets of genes, or pathways, that are thought to have coordinated effects on a biological endpoint.

A number of pathway analysis methods have been developed, and have been widely applied in human genetics and genomics. The majority of pathway analysis methods were originally developed for microarray, gene expression data, and the most popular methods perform enrichment analysis for gene sets defined by external knowledge bases [11]. In the current study, we modified the bioinformatics approaches that have been developed in other contexts to be valid for epigenome-wide data analysis.

Importantly, we performed a two stage study, performing both discovery and replication of the gene-based and pathway-based associations. While replication is standard in genetic association studies for individual variants it is rarely performed for pathway analyses. Whether due to the limited availability of proper validation cohorts in many studies, or challenges in adapting pathway approaches to allow for a discovery and replication approach, this lack of replication is an important limitation of many pathway analysis studies. The previously described MoBa cohort, referred to as MoBa1 was used as the discovery cohort. We subsequently measured DNA methylation in an additional 685 MoBa newborns; this dataset is referred to as MoBa2 and is used as the replication cohort.

## Results

**Fig. 2:**
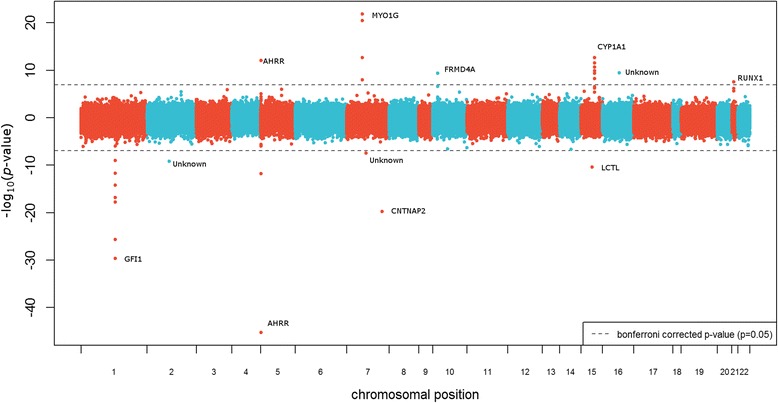
Manhattan plot of univariate CpG results. The y-axis represents the –log10 of the CpG *p*-values. CpGs with negative *p*-values corresponded to decreased methylation, whereas positive *p*-values corresponded to increased methylation. CpGs that reached genome-wide significance, with a bonferonni corrected *p* < 0.05 are annotated with their corresponding genes

In univariate analysis of individual CpGs in the discovery cohort MoBa1, we found methylation at 27 CpGs in newborns to be significantly associated with maternal plasma cotinine levels analyzed as a continuous variable (Bonferroni correction for 473,864 tests, *p* < 1.06 × 10^−7^). The majority of those markers are annotated within genes. Twenty four markers are annotated within the *GFI1*, *AHRR*, *MYO1G*, *CNTNAP2*, *FRMD4A*, *LCTL*, *CYP1A1*, and *RUNX1* genes (Fig. 2). The three significant markers (cg00253658, cg18703066, cg04598670) that did not map to known genes are located on chr16 at 54210496, chr2 at 105363536, and chr7 at 68697651.

We then grouped individual CpGs by gene to form a gene-level *p* value, or gene score, using the Sequence Kernel Association Test (SKAT) software implemented in R [12, 13]. A total of 20,199 genes were tested and 15 were associated with maternal plasma cotinine levels with an FDR-adjusted *q* < 0.25 (Table 1). Six of these 15 genes (*GFI1*, *MYO1G*, *CYP1A1*, *RUNX1*, *LCTL*, and *AHRR*) contained genome-wide significant individual CpGs (*p* < 1.06 × 10^−7^). Nine of the 15 genes (*FCRLA*, *MIR641*, *SLC25A24, TRAK1*, *C1orf180*, *ITLN2*, *GLIS1*, *LRFN1*, and *MIR451*) were associated with cotinine (*q* < 0.25) but did not have any genome-wide significant individual CpGs (Table 1). This demonstrates the utility of this method to detect important effects at a gene-level that would have otherwise gone undetected by interrogating only individual CpGs.

**Table 1:** Genes differentially methylated in newborns in relation to maternal smoking during pregnancy using the Sequence Kernel Association Test (SKAT) in the MoBa1 discovery cohort (*n* = 1062 subjects)

Gene^a^	Markers/Gene	SKAT *p*-value	SKAT *q*-value
*GFI1*	71	1.05E-17	2.13E-13
*MYO1G*	12	4.33E-17	4.37E-13
*CYP1A1*	35	1.21E-09	8.15E-06
*RUNX1*	53	3.46E-07	0.001749
*LCTL*	8	1.61E-05	0.065098
*AHRR*	149	6.29E-05	0.184672
*FCRLA*	9	8.14E-05	0.184672
*MIR641*	4	8.23E-05	0.184672
*TRAK1*	35	7.78E-05	0.184672
*C1ORF180*	4	0.000104	0.209611
*ITLN2*	5	0.000116	0.212334
*GLIS1*	51	0.000156	0.223673
*LRFN1*	21	0.00016	0.223673
*MIR451*	8	0.000166	0.223673
*SLC25A24*	23	0.000144	0.223673

^a^ Covariates included: maternal education, CD8T, CD4T, natural killer cell fraction, B cell fraction, monocyte fraction, granulocyte fraction

Only two genes, *CNTNAP2* and *FMRD4A*, had genome-wide significant individual CpGs (*p* < 1.06 × 10^−7^), but did not result in gene scores with *q* < 0.25. Eighty CpGs mapped to *CNTNAP2* but only one (cg25949550), located in the gene body, was statistically significant (*q* = 1.07 × 10^−13^) resulting in a gene score (*q* = 0.32) that did not reach our threshold for association (Additional file 1). There were 127 CpGs mapped to *FRMD4A* on this platform and only two CpGs (cg11813497, cg15507334), located within 200 bp of the transcriptional start site, were at or near genome-wide significance, for an overall gene score with a *q* = 0.28 (Additional file 1).

We then collapsed the gene-level results into pathway level statistics using *a priori* pathway gene sets from the MSigDB database. MSigDB provides annoted collections of gene sets curated from multiple biological knowledge-bases. We selected relevant gene sets as described below to collapse individual gene association scores into pathway analysis results. A total of 5836 pathway gene sets were tested for association using a the correlated Lancaster *p*-value approach. After a Bonferroni correction (*p* < 0.05) for the number of pathways tested, a total of 51 pathways were statistically significant in the (Fig. 1 and Table 2). Pathways spanned a range of physiological and pathophysiological functions including cell cycle, cancer, white blood cell differentiation, genotoxicity, and others (Additional file 2).

**Table 2:** Significantly enriched pathways based on differential methylation in newborns exposed to maternal smoking during pregnancy

Pathway Name	MSigDB Contributor^a^	MSigDB Category Code	# Genes Pathway	# Genes Overlap	Discovery *p* value	Bonferroni Adjusted Discovery *p* value	Replication *p* value	Replication *q* value	Bonferroni Adjusted Replication *p* value
GSE17974_0H_VS_12H_IN_VITRO_ACT_CD4_TCELL_UP	Nick Haining lab (DFCI)	C7	200	172	1.56E-14	9.12E-11	9.31E-07	2.60E-05	4.28E-05
GSE17974_CTRL_VS_ACT_IL4_AND_ANTI_IL12_2H_CD4_TCELL_UP	Nick Haining lab (DFCI)	C7	200	171	3.01E-14	1.76E-10	1.13E-06	2.60E-05	5.19E-05
GSE17974_0H_VS_6H_IN_VITRO_ACT_CD4_TCELL_UP	Nick Haining lab (DFCI)	C7	200	169	1.55E-17	9.07E-14	5.33E-06	8.17E-05	0.0002
G1_S_TRANSITION_OF_MITOTIC_CELL_CYCLE	GO	C5	27	27	1.16E-18	6.74E-15	0.0012	0.0135	0.0548
WILLIAMS_ESR1_TARGETS_DN	Broad Institute	C2	6	6	1.83E-08	0.000107	0.0015	0.0135	0.0675
TIEN_INTESTINE_PROBIOTICS_2HR_UP	Broad Institute	C2	27	26	2.54E-08	0.000148	0.0019	0.0144	0.0862
TONKS_TARGETS_OF_RUNX1_RUNX1T1_FUSION_SUSTAINED_IN_MONOCYTE_UP	Broad Institute	C2	21	21	6.48E-09	3.78E-05	0.0027	0.0158	0.1226
INTERPHASE_OF_MITOTIC_CELL_CYCLE	GO	C5	62	59	1.17E-16	6.85E-13	0.0027	0.0158	0.1264
HEDENFALK_BREAST_CANCER_BRACX_UP	University of Washington	C2	20	14	4.81E-10	2.80E-06	0.0032	0.0159	0.1464
ABE_VEGFA_TARGETS_2HR	University of Washington	C2	34	30	2.16E-09	1.26E-05	0.0036	0.0159	0.1675
INTERPHASE	GO	C5	68	65	7.20E-17	4.20E-13	0.0038	0.0159	0.1753
FRASOR_RESPONSE_TO_ESTRADIOL_UP	Broad Institute	C2	37	37	3.72E-08	0.000217	0.0046	0.0175	0.2099
ENGELMANN_CANCER_PROGENITORS_UP	Broad Institute	C2	48	47	4.18E-07	0.002441	0.0059	0.0209	0.2713
MIKKELSEN_IPS_WITH_HCP_H3K27ME3	Broad Institute	C2	102	97	4.80E-13	2.80E-09	0.0073	0.0239	0.3346
FALVELLA_SMOKERS_WITH_LUNG_CANCER	Broad Institute	C2	80	71	5.90E-08	0.000344	0.0091	0.0278	0.4165
AMUNDSON_GENOTOXIC_SIGNATURE	Broad Institute	C2	105	94	1.22E-15	7.15E-12	0.0110	0.0305	0.5045
GSE1460_DP_VS_CD4_THYMOCYTE_UP	Nick Haining lab (DFCI)	C7	200	174	1.29E-16	7.53E-13	0.0113	0.0305	0.5186
GSE3982_DC_VS_TH1_DN	Nick Haining lab (DFCI)	C7	200	174	4.75E-18	2.77E-14	0.0124	0.0307	0.5702
ELVIDGE_HIF1A_TARGETS_DN	Broad Institute	C2	91	85	2.82E-08	0.000165	0.0127	0.0307	0.5824
CASORELLI_ACUTE_PROMYELOCYTIC_LEUKEMIA_UP	Broad Institute	C2	177	150	9.14E-14	5.34E-10	0.0140	0.0322	0.6444
GSE1460_DP_THYMOCYTE_VS_NAIVE_CD4_TCELL_ADULT_BLOOD_UP	Nick Haining lab (DFCI)	C7	200	170	3.93E-19	2.29E-15	0.0164	0.0353	0.7547
GSE17974_CTRL_VS_ACT_IL4_AND_ANTI_IL12_2H_CD4_TCELL_DN	Nick Haining lab (DFCI)	C7	200	181	1.27E-16	7.42E-13	0.0172	0.0353	0.7901
GSE22886_NAIVE_CD4_TCELL_VS_48H_ACT_TH2_DN	Nick Haining lab (DFCI)	C7	200	183	1.11E-15	6.48E-12	0.0194	0.0353	0.8933
GSE24634_NAIVE_CD4_TCELL_VS_DAY10_IL4_CONV_TREG_DN	Nick Haining lab (DFCI)	C7	200	185	5.91E-15	3.45E-11	0.0195	0.0353	0.8948
GSE3982_CENT_MEMORY_CD4_TCELL_VS_TH1_DN	Nick Haining lab (DFCI)	C7	200	185	1.44E-14	8.38E-11	0.0195	0.0353	0.8973
GSE17974_0H_VS_4H_IN_VITRO_ACT_CD4_TCELL_DN	Nick Haining lab (DFCI)	C7	200	182	1.43E-14	8.32E-11	0.0205	0.0353	0.9451
GSE3982_EOSINOPHIL_VS_TH1_DN	Nick Haining lab (DFCI)	C7	200	189	7.96E-15	4.65E-11	0.0209	0.0353	0.9599
GSE3982_NEUTROPHIL_VS_TH1_DN	Nick Haining lab (DFCI)	C7	200	182	2.96E-16	1.73E-12	0.0215	0.0353	0.9897
GSE24634_NAIVE_CD4_TCELL_VS_DAY7_IL4_CONV_TREG_DN	Nick Haining lab (DFCI)	C7	200	189	1.20E-15	7.00E-12	0.0250	0.0388	1
GSE15215_CD2_POS_VS_NEG_PDC_DN	Nick Haining lab (DFCI)	C7	200	180	3.26E-17	1.90E-13	0.0253	0.0388	1
GSE10856_CTRL_VS_TNFRSF6B_IN_MACROPHAGE_DN	Nick Haining lab (DFCI)	C7	200	170	1.47E-06	0.008573	0.0274	0.0407	1
GSE3982_MAC_VS_TH2_DN	Nick Haining lab (DFCI)	C7	200	182	5.98E-08	0.000349	0.0296	0.0426	1

^a^ Contributor to the corresponding pathway in MSigDB. Additional information about these contributors can be found at: http://www.broadinstitute.org/gsea/msigdb/collection_details.jsp

Subsequently, we attempted to replicate the pathway analysis by calculating gene scores in the MoBa2 replication cohort data for all genes in the 51 statistically significant pathways from the MoBa1 discovery cohort. Gene and pathway level association scores were calculated identically to the procedure described for the discovery cohort (Fig. 1), and a FDR correction was used to correct for multiple testing. Of the 51 pathways identified in the MoBa1 cohort (*p* < 8.6 × 10^−6^), 32 replicated (*q* < 0.05) (Table 2).

**Fig. 3:**
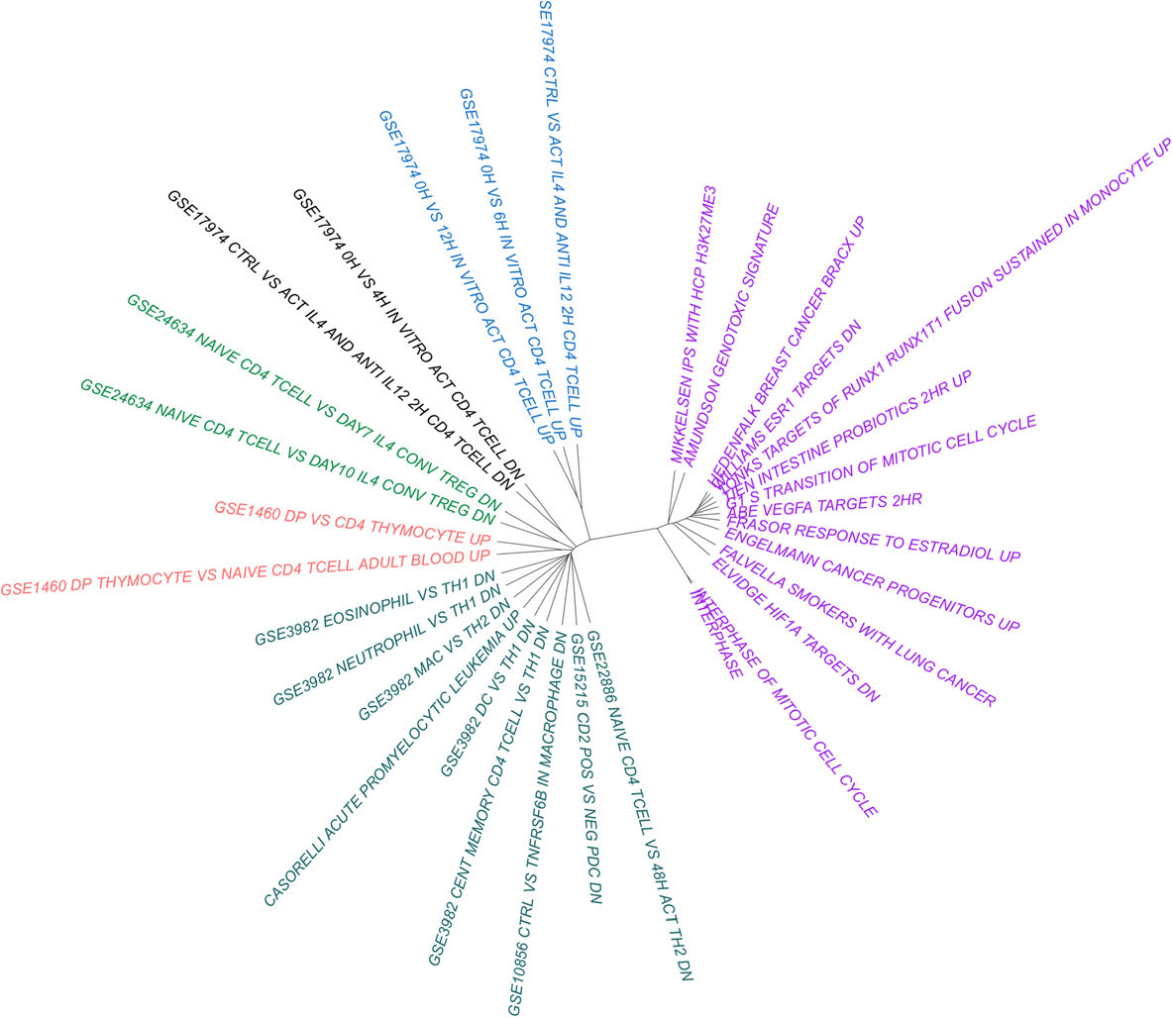
Hierarchical clustering of replicated pathways. Replicated pathways were measured for similarity and clustered based on overlapping genes between each pathway. The dendrogram was cut to show six distinct clusters; pathways within the same cluster are annotated with matching colors

Because of the relatively large number of pathways that replicated across both cohorts, we performed clustering analysis to aid in interpretability. We clustered replicated pathways according to gene set similarity (Fig. 3). We identified six clusters, or groups, of pathways that contained similar gene sets and were reflective of their biological function. The largest cluster consisted of pathways related to cancer (FALVELLA SMOKERS WITH LUNG CANCER, HEDENFALK BREAST CANCER BRACX UP), cell cycle (INTERPHASE OF MITOTIC CELL CYCLE, INTERPHASE, G1 S TRANSITION OF MITOTIC CELL CYCLE), ERα receptor signaling (WILLIAMS ESR1 TARGETS DN, FRASOR RESPONSE TO ESTRADIOL UP), and angiogenesis (ABE VEGFA TARGETS 2HR, ELVIDGE HIF1A TARGETS DN). A second cluster was organized into five smaller pathway groups related to immune system function, such as T-cell regulation (e.g. GSE1460 DP THYMOCYTE VS NAIVE CD4 TCELL ADULT BLOOD UP, GSE3982 DC VS TH1 DN, GSE3982 CENT MEMORY CD4 TCELL VS TH1 DN) and other white blood cell related pathways (e.g. GSE1460 DP VS CD4 THYMOCYTE UP, CASORELLI ACUTE PROMYELOCYTIC LEUKEMIA UP).

## Discussion

There is an overwhelming body of epidemiological evidence linking smoking during pregnancy to various health outcomes in the offspring including low birth weight, reduced lung function, and increased respiratory infections [1]. Additional associations have also been reported between maternal smoking during pregnancy and 1) rheumatoid arthritis and other inflammatory polyarthropathies [14–17], 2) child behavior and cognitive functioning, and 3) mixed results of associations with childhood cancers. While these associations are consistent, the underlying mechanisms leading to these outcomes have remained elusive. The analyses presented here support the possibility that epigenetic mechanisms may play a role, and point towards a number of pathways that may be involved.

Multiple pathways related to T-cell function were altered by maternal smoking. GFI1, previously reported by Joubert et al. [3], was a main driver for many of the T-cell, eosinophil, and neutrophil related pathway scores (e.g. GSE17974_0H_VS_12H_IN_VITRO_ACT_CD4_TCELL_UP, GSE3982_CENT_MEMORY_CD4_TCELL_VS_TH1_DN, GSE3982_NEUTROPHIL_VS_TH1_DN, GSE3982_EOSINOPHIL_VS_TH1_DN). Additional genes that contributed to the impact on immune response pathways include *IL22* (*p* = 0.039, *q* = 0.28) and *IL2RA* (*p* = 0.002, *q* = 0.28) which were not detected in the analysis of Joubert et al. [3] based on single CpGs.

*IL22* is a cytokine involved in the initiation of innate immune response against pathogens, and is especially active in epithelial cells of the gut and lung [18]. Reduced expression of *IL2RA* on the surface of immune cells has been known to cause chronic immune suppression and may be linked to type 1 diabetes mellitus [19, 20]. Collectively, these pathways are relevant to various health effects in newborns that have been associated with exposure to maternal smoking during pregnancy [14, 17, 21].

Mixed results have been found regarding in utero tobacco exposure and increased incidence of childhood cancers. Some studies have found increased risk of childhood cancers with maternal smoking during pregnancy [16, 22], whereas, others have found null results [15, 23]. However, here we present evidence that alterations in methylation may affect key pathways related to cancer. Joubert et al. [24] demonstrated that maternal smoking affects newborn methylation if the mother smokes through gestational week 18, whereas significant effects on methylation were not observed for mothers that quit before 18 gestational weeks. Some studies assessed smoking during pregnancy as any smoking versus no smoking. Thus if sustained smoking during pregnancy is required, as suggested by the methylation analyses, associations with cancer might be attenuated or missed entirely.

In addition to cancer-specific pathways (i.e. HEDENFALK_BREAST_CANCER_BRACX_UP, ENGELMANN_CANCER_PROGENITORS_UP, FALVELLA_SMOKERS_WITH_LUNG_CANCER, CASORELLI_ACUTE_PROMYELOCYTIC_LEUKEMIA_UP), changes in pathways related to cell cycle were detected, which are also relevant to cancer (i.e. G1_S_TRANSITION_OF_MITOTIC_CELL_CYCLE, INTERPHASE_OF_MITOTIC_CELL_CYCLE). These pathway level effects were also mainly driven by *GFI1*.

However, decreased methylation of the gene Speedy (*SPDYA*) (*p* = 0.024, *q* = 0.28) also contributed to the impact on INTERPHASE_OF_MITOTIC_CELL_CYCLE. *SPDYA* was not identified in the analysis of individual CpGs by Joubert et al. [3]. It is a cell cycle regulator that has been shown to increase cell proliferation through activation of cyclin dependent kinase-2 (cdk2) during the G1/S phase of cellular replication [25]. The ABE_VEGFA_TARGETS_2HR pathway, related to vascular endothelial growth factor-A gene (*VEGFA*), was significantly altered (replication *q* = 0.03). *VEGFA* mediates angiogenesis, suppresses apoptosis, and is the pharmacological target for Bevacizumab, a monoclonal antibody chemotherapeutic drug [26–28]. *VEGFA* is increased during oxidative stress and results in a compensatory increase in angiogenesis, a hallmark of cancer [28–30].

Furthermore, impacts on pathways WILLIAMS_ESR1_TARGETS_DN and FRASOR_RESPONSE_TO_ESTRADIOL_UP point towards effects related to estrogen receptor-alpha (ERα) signaling which is important in several cancers [31–33]. Effects on these pathways were largely mediated through CYP1A1 (*p* = 1.21 × 10^−9^), which was previously identified by Joubert et al., and PDZK1 (*p* = 0.0007) which was not.

Effects on pathways related to cell cycle and angiogenesis may also point towards mechanisms by which birth weight may be affected. Recently, a study by Miller et al. [34] demonstrated a differential effect on male birth weight from non-smoking mothers if the maternal grandmother smoked while pregnant, suggesting a potential epigenetic mechanism may be responsible. Decreased birth weight is a well-established effect of maternal smoking on offspring, although the mechanism by which this occurs has not been elucidated [35].

Through the novel implementation of methods for creating gene scores [13] and pathway scores [36], we have identified and replicated key biological processes related to maternal smoking via its impact on newborn DNA methylation. These methods permit replication, which limits the likelihood of false-positive findings. To our knowledge, until now no studies of pathway impacts on methylation have been performed in tandem with a replication dataset. Furthermore, using gene based tests, we identified associations with genes not identified by CpG specific analyses alone – these included *FCRLA*, *MIR641*, *SLC25A24, TRAK1*, *C1orf180*, *ITLN2*, *GLIS1*, *LRFN1*, and *MIR451*.

The replicated pathway analysis conducted offers potential new insights into the biological impacts of maternal smoking on fetal DNA methylation. The genes and pathways detected point to effects on T-cell mediation, cell cycle, and xenobiotic metabolism. In turn, these data further support a potential epigenetic role for the adverse health effects observed in children exposed to maternal smoking during pregnancy.

## Methods

### Study population

Participants in this analysis include 1062 mother-offspring pairs from a substudy of the Norwegian Mother and Child Cohort Study (MoBa) [37–39]. In a previous study with this cohort, individual CpG sites in newborns were tested for differential methylation in relation to maternal smoking [3]. This dataset is referred to as MoBa1 and was used as the discovery cohort. We subsequently measured DNA methylation in an additional 685 newborns. This dataset is referred to as MoBa2 and was used as the replication cohort. The study has been approved by the Regional Committee for Ethics in Medical Research, the Norwegian Data Inspectorate and the Institutional Review Board of the National Institute of Environmental Health Sciences, USA, and written informed consent was provided by all mothers participating.

### Covariates and cotinine measurements

Information on maternal age, parity, and maternal education was collected from questionnaires completed by the mother or from birth registry records. Maternal age was included as a continuous variable. Parity was categorized as 0, 1, 2, or >=3 births. Maternal educational level was categorized as previously described Joubert et al. [3], indicative of less than high school/secondary school, high school/secondary school completion, some college or university, and 4 years of college/university or more. Maternal smoking during pregnancy (none, stopped before 18 weeks of pregnancy, smoked past 18 weeks of pregnancy) was assessed by maternal questionnaire and verified with maternal plasma cotinine measured by liquid chromatography - tandem mass spectrometry at approximately 18 weeks gestation [40].

For MoBa1, cotinine, a quantitative biomarker of smoking, was measured in maternal plasma and was analyzed as a continuous variable. No cotinine was detected in 736 participants, and of the participants with detectable cotinine levels (*N* = 326) the mean cotinine level was 191 (SE = 11). For MoBa2, cotinine measurements were not available for most mothers. Therefore, a three-category variable based on the mother’s report of smoking during pregnancy was created and supported using cotinine measurements when available (*N* = 221 MoBa2 participants had cotinine data). The three categories represented no smoking (*N* = 512), stopped during pregnancy (*N* = 103), or smoked throughout pregnancy (*N* = 70).

### Methylation measurements

Details of the DNA methylation measurements and quality control for the MoBa1 participants were previously described [3] and the same reagents, platforms and protocols were used for the MoBa2 participants. All biological material was obtained from the Biobank of the MoBa study [38]. Briefly, DNA was extracted from umbilical cord whole blood samples [36]. Bisulfite conversion was performed using the EZ-96 DNA Methylation kit (Zymo Research Corporation, Irvine, CA) and DNA methylation was measured at 485,577 CpGs in cord blood using Illumina’s Infinium HumanMethylation450 BeadChip [41, 42]. The package *minfi* in R was used to calculate the methylation level at each CpG as the beta-value (β = intensity of the methylated allele (M)/(intensity of the unmethylated allele (U) + intensity of the methylated allele (M) + 100)) from the raw intensity (idat) files [43, 44].

Probe and sample-specific quality control filtering was performed separately in MoBa1 and MoBa2 datasets. Control probes (*N* = 65) and probes on X (*N* = 11,230) and Y (*N* = 416) chromosomes were excluded in both datasets. Remaining CpGs missing >10% of methylation data were also removed (*N* = 20 in MoBa1, none in MoBa2). Samples indicated by Illumina to have failed or have an average detection *p*-value across all probes < 0.05 (*N* = 49 MoBa1, *N* = 35 MoBa2) and samples with gender mismatches (*N* = 13 MoBa1, *N* = 8 MoBa2) were also removed. For each dataset, we accounted for the two different probe designs by applying the intra-array normalization strategy Beta Mixture Quantile dilation (BMIQ) [45].

The gPCA program was used to determine the presence of batch effects, using plate to represent batch and ComBat was applied for batch correction using the SVA package in R for both MoBa 1 and MoBa 2 cohorts [44, 46–48]. A total of 473,772 markers remained after data processing, and 365,860 of these markers mapped to at least one of 21,231 genes using Illumina provided annotation based on human reference genome [NCBI build 37].

### Covariate selection

All analysis was conducted in the statistical programming language, R [44]. Initially, potential clinical and demographic variables: maternal age, newborn gender, education, asthma, folate, and parity were evaluated as potential covariates prior to association analysis. Each potential covariate was tested for association with maternal cotinine using linear least squares regression, with categorical variables dummy encoded in the model(s). Two-sided *p*-values from each regression analysis were recorded, and a False Discovery Rate (FDR) correction for multiple comparisons was applied to limit false positives. Covariates with an FDR-adjusted *q* value < 0.1 were included in subsequent models [49]. In addition, cell type fractions (CD8T, CD4T, natural killer cell, B cell, monocyte, granulocyte) for each subject were calculated using the reference-based Houseman method in the *minfi* package in *R* [43, 44, 50], and these fractions were forced as covariates into subsequent models. The same selection criteria was used for both the discovery and replication dataset. The only resulting covariate was maternal education for MoBa1 (*q* < 0.1), and maternal age, education, folate, and parity were selected as covariates for MoBa2 (*q* < 0.1).

### Univariate association analysis

Statistical tests for the association of each CpG marker and maternal plasma cotinine levels (continuous) were performed using linear least-squares regression for the MoBa1 cohort. Significant covariates and cell type fractions were included in the model to reduce confounding. All CpG *p* values, on the -log_10_ scale, were plotted according to genomic sequence in a Manhattan plot (Fig. 1).

### Gene score calculation

To perform gene-level association analysis, CpG markers were collapsed by gene using the Illumina provided annotation based on human reference genome [NCBI build 37]. For each gene, the CpG data was combined into a gene-level *p* value using the Sequence Kernel Association Test (SKAT) software implemented in R [12, 13]. The SKAT null model for MoBa1 was created using significantly associated covariates: maternal education (*q* < 0.1), and cell type fractions (CD8T, CD4T, natural killer cell, B cell, monocyte, granulocyte). The same modeling strategy was implemented for the SKAT null model for MoBa2 and included significantly associated covariates and the cell type fractions. The SKAT model was then run using an unweighted, linear kernel with the ‘is_check_genotype’ flag set to FALSE. In order to account for the underlying correlation structure for the *p* value gene scores, the SKAT null model was created with the cotinine values and covariate values randomly shuffled, and then SKAT was run on the residuals until 1000 permuted gene scores were created. To control for multiple comparisons, we report gene scores with a FDR *q* < 0.25 as being associated with cotinine levels.

### Pathway analysis

The results from the SKAT gene-level association analysis (specifically *p*-values) were used for pathway-level analysis. Genes were grouped into a priori pathways (gene sets) using the Molecular Signatures Database v4.0 (MSigDB) [51]. MSigDB contains gene sets from a collection of popular resources such as Gene Ontology (GO) and the Kyoto Encyclopedia of Genes and Genomes (KEGG) [51]. A subset of pathways was selected for analysis based on a set of four criteria: 1) the pathway must be composed of a set of genes from *Homo sapiens,* 2) the number of genes in a pathway cannot exceed 250 genes, 3) at least one gene in the pathway must be present in the list of available gene scores, and 4) pathways representing positional gene sets (C1), motif gene sets (C3), and computational derived gene sets (C4) were excluded. This resulted in a total of 5836 pathways for analysis. These pathways came from the either curated gene sets (C2), GO gene sets (C5), oncogenic signatures gene sets (C6), or the immunologic signatures gene sets (C7) collections in MSigDB [9]. Each pathway consists of a set of genes that are considered biologically relevant to a given biological function or signaling network, and individual genes are often represented in multiple pathways.

The pathway-level score was calculated from the individual gene scores that overlapped with the genes in each pathway gene set. The pathway level score is the combined *p*-value across all gene-level results from the SKAT analysis. There are a number of approaches for combining *p*-values, but most assume that the individual *p*-values are not correlated. Pathway analysis actually relies on the fact that genes scores within a pathway are correlated, so a collapsing approach that explicitly takes that into account was used. More specifically, the individual gene scores were combined into pathway-level scores using the correlated Lancaster method in Dai et al. (T_A_) [36]. This resulted in a final *p*-value for each pathway from MSigDB. It is important to note that this combined *p*-value represents a self-contained pathway analysis, where the null hypothesis is that gene sets are not more strongly associated than expected by chance. Because of the large number of pathways tested, we controlled for multiple comparisons using a conservative Bonferroni correction. We chose a conservative approach, even though the *p*-values from each pathway are not independent, since genes appear in multiple pathways. Pathways with a corrected *p* < .05 (*n* = 5836; *p* < 8.6 × 10^−6^) were considered statistically significant in the discovery cohort.

### Replication

The statistically significant pathways (*p* < 8.6 × 10^−6^) were tested for replication using MoBa2. The CpG values were combined for genes that occurred in significant pathways in MoBa1, using SKAT as described above. Gene scores were then combined using the Lancaster approach to calculate a pathway-level score for the replication cohort. Pathways *p* values were adjusted using both an FDR and a more conservative Bonferroni approach and were considered to be successfully replicated with an FDR *q* < 0.05 (Table 2). Pathway analyses are commonly divided into self-contained or competitive approaches. Here we use a self-contained, global null approach to pathway analysis. An advantage of this approach is that it lends itself toward replication in smaller cohorts because only genes in significant pathways from the discovery cohort need to be tested for replication. Competitive pathway analysis methods test a different null hypothesis, and subsequently require all genes to be tested, which can make replication with smaller cohorts unfeasible.

### Pathway hierarchical clustering

Hierarchical clustering was performed using R and the ‘APE’ package [44, 52]. All unique genes within replicated pathways (*q* < .05) were tabulated. All gene-pathway combinations were recorded as either a “1” if the pathway contained the gene or a “0” if the pathway did not contain the gene. Clustering was then performed using Euclidean distance and Ward’s method. The resulting dendrogram (Fig. 3) was then cut and colored so that six groups were defined based on gene set similarity.

## Conclusions

We used a novel implementation of bioinformatics tools to collapse individual CpG results to a gene score and performed pathway analysis to test for in utero epigenetic changes by maternal smoking in 1062 participants in the MoBa. By collapsing individual CpG effects to gene scores, we found significant differential methylation in 15 genes (q<0.25), nine of which were not detected by only testing individual CpGs. Furthermore, pathway analysis revealed significant associations with 51 pathways, 32 of which replicated in an independent cohort of 685 participants. Significantly associated pathways, that replicated in the independent cohort, represent diverse biological processes including cancer, cell cycle, ERα receptor signaling, angiogenesis, and immune system function. This approach may provide new insight into the biological mechanisms that may lead to adverse health effects from exposure to tobacco smoke in utero.

## Additional files

Additional file 1: SKAT_GeneScor. (XLSX 1 MB)
Additional file 2: Lancaster_Pat. (XLSX 4 MB)

## Abbreviations

BMIQ: Beta Mixture Quantile dilation
cdk2: cyclin dependent kinase-2
CpGs: Region where cytosine and guanine are separated by one phosphate. The cytosine at these sites can be methylated
FDR: False Discovery Rate
GO: Gene Ontology
GSEA: Gene Set Enrichment Analysis
KEGG: Kyoto Encyclopedia of Genes and Genomes
MoBa: Norwegian Mother and Child Cohort Study
MSigDB: Molecular Signatures Database v4.0
SKAT: Sequence Kernel Association Test
*SPDYA*: Speedy gene
*VEGFA*: Vascular endothelial growth factor-A gene
